# Experiences of Participant Burden in an Alzheimer’s Disease Research Center Longitudinal Cohort

**DOI:** 10.1002/alz.095026

**Published:** 2025-01-09

**Authors:** Theresa F. Gierzynski, Jonathan M Reader, Bernardo Flores, Renee Gadwa, Arijit K Bhaumik, Annalise Rahman‐Filipiak, Judith L Heidebrink, Bruno Giordani, Benjamin M. Hampstead, Kelly M. Bakulski, Henry L Paulson, J. Scott Roberts

**Affiliations:** ^1^ University of Michigan, Ann Arbor, MI USA; ^2^ Michigan Alzheimer’s Disease Research Center, Ann Arbor, MI USA; ^3^ Michigan Alzheimer’s Disease Research Center ‐ University of Michigan, Ann Arbor, MI USA; ^4^ Michigan Alzheimer’s Disease Center, Ann Arbor, MI USA

## Abstract

**Background:**

Longitudinal studies of Alzheimer’s and related dementias (ADRD) are crucial to understanding disease progression. Retention among study participants is vital to successful longitudinal research, as dropout can skew the subject population in ways that significantly compromise study outcomes (e.g., limited generalizability). Prior research suggests that higher perceptions of burden among research volunteers are associated with an increased likelihood of attrition and missed visits. Additionally, underrepresented groups (e.g., racial minorities) may be uniquely impacted by barriers that hinder participation and sustained engagement in ADRD research. The current study aimed to measure participants’ perceived burden in a cohort of longitudinal ADRD research participants and assess for potential differences in perceived burden by race.

**Methods:**

Participants enrolled in a study of memory and aging at the Michigan Alzheimer’s Disease Research Center (MADRC) were administered a modified, 22‐item version of the Perceived Research Burden Assessment (PeRBA) survey instrument (Keleman et al., 2023) either in‐person or via telephone. The PeRBA is a validated measure assessing overall, logistical, psychological, and physical burdens of participating in research. A series of Wilcoxon Rank Sum Tests were performed to assess for possible racial differences in the mean scores of overall burden, as well as in each subscale.

**Result:**

A total of 76 participants completed the PeRBA; the sample was 18.8% Black/African American, 67.1% female, and 55.3% cognitively unimpaired. Overall burden ranged from 21 to 55 (110 possible) (Overall Mean (SD) = 36.23 (8.25); Black/African American Mean (SD) = 43.86 (2.07); White Mean (SD) = 34.61 (8.17)). Results indicated that Black/African American participants experienced significantly more overall burden than their White counterparts (all *p*s < 0.05) as well as across all subscales.

**Conclusion:**

Further research is needed to better understand the ostensibly higher appraisals of research burden in our Black/African American participants. Future investigations should consider the role of geographic/site‐specific factors as well as structural (e.g., socioeconomic status, transportation) and psychosocial influences and their role in participant retention and recruitment. Moreover, the identification of deterrents and barriers to engagement can inform tailored strategies that support the specific needs of diverse older adult populations in ADRD studies.

